# Level of Agreement Between a Modified, Three-Step Menstrual Cycle Tracking Method and a Female-Health Menstrual Cycle Tracking App

**DOI:** 10.1177/26884844251379415

**Published:** 2025-09-30

**Authors:** Marissa L. Doroshuk, Constance M. Lebrun, Patricia K. Doyle-Baker

**Affiliations:** ^1^ Human Performance Lab, Faculty of Kinesiology, University of Calgary, Calgary, Alberta, Canada.; ^2^ Faculty of Medicine and Dentistry, University of Alberta, Edmonton, Alberta, Canada.; ^3^ Alberta Children’s Hospital Research Institute, University of Calgary, Calgary, Alberta, Canada.; ^4^ O'Brien Institute for Public Health, University of Calgary, Calgary, Alberta, Canada.

**Keywords:** ovulation, menstrual cycle tracking, mobile application, limits of agreement, mHealth

## Abstract

**Background:**

A need exists to incorporate evidence-based tracking methods that measure menstrual cycle (MC) variability to describe the data quality provided by an app. The study purpose was to assess the agreement between an app’s cycle phase identifications and a modified version of the three-step method (m3stepMC) of hormone verification.

**Materials and Methods:**

Participants across Canada were recruited to track their MC over 3 months by entering data into a female-health MC tracking app (the app) while collecting measures of ovulation and salivary hormones around the late-follicular (FP) and mid-luteal (MLP) phases, respectively. Bland–Altman plots assessed the limits of agreement (LoA) between the identified days within each of the app’s predetermined phases and the m3stepMC-identified days when MC dates aligned between the methods. Pearson’s correlations (*r*) were used to examine the effect size of relationships between variables.

**Results:**

Participants’ (*n* = 25) mean age was 29.3 ± 4.24 with self-reported mean cycle lengths of 27.3 ± 2.38 days. The agreement between the app’s estimated (1) end of phase one and the estimated start of the mid-FP was 0.6 ± 1.66 days (95% LoA: 2.65–3.85; *r* = 0.66), (2) end of phase two and the identified luteinizing hormone (LH) surge day and midpoint of phase three and the estimated 48-hour ovulatory window post-LH surge day were −0.6 ± 1.71 days (95% LoA: −3.95 to 2.75; *r* = 0.64), and (3) phase four and the estimated MLP day verified by salivary hormones and the start of the app’s phase five and the estimated late-luteal midpoint day were −2.2 ± 0.97 (95% LoA: −4.13 to −0.32; *r* = 0.94).

**Conclusion:**

This study describes the agreement between a m3stepMC tracking method and hormone measures and an app’s predetermined MC phase system in eumenorrheic cycles when MC dates aligned between methods.

## Introduction

The menstrual cycle (MC) includes several phases that can range from two to six when considering the follicular phase, ovulatory or fertile window, luteal phase, and their subcategories of early, mid, and late.^1,2^ Apps that track the MC use a variety of predetermined classifications that employ algorithms to identify cycle length, symptoms, and phases, with many emphasizing the ovulatory window for fertility tracking.^3,4^ However, as menstrual tracking apps have evolved, some of these apps now emphasize female-focused health data collection to understand MC variability.^5^ Previous research has recommended using prospective tracking methods that employ a combination of steps over several cycles to determine menstrual phases.^2,6,7^ Cycle length can be variable between individual cycles, which subsequently impacts the timing of menstrual phases.^8–10^ Current methods include the direct measurements of luteinizing hormone (LH), estradiol, and progesterone levels for identification of the cycle phase.^6,11–14^

A practical approach for identifying the mid-luteal menstrual phase includes a combination of MC mapping or calendar counting, urinary ovulation testing for LH surge detection, and verification of hormones through serum methods. An approach that uses this combination in the literature is the three-step method by Schaumberg et al.^2^ However, in recent years, salivary hormone analysis in MC studies has become a complementary hormone verification method to serum due to its simplicity and rapid assessment of steroid sex hormones.^15,16^

In summary, apps that track the MC have increased in use.^3,4,17^ A need exists to describe the data provided by an app relative to a menstrual tracking method that can identify individual cycle variability through ovulation and hormone measurements.^3,4^ Therefore, the purpose of this study was to assess the level of agreement (agreement) between a modified, three-step MC (m3stepMC) tracking method and the five predetermined MC phases in a female-health MC tracking app (the app).

## Materials and Methods

### Design and recruitment

This prospective observational cohort study employed a rolling recruitment from October 2022 to April 2023, with data collection ending in September 2023. Participants were recruited primarily through University of Calgary social media sites, as well as posters placed around campus, clinics, fitness facilities, and snowball sampling. Participant involvement varied between 3 and 6 months, with 1 month for each aspect of the m3stepMC tracking method and continued up to 3 months if warranted to account for missing data. An informed consent was completed prior to the study, and the study began with the participant’s following menstruation. Approval for the study was received from the Conjoint Health Research Ethics Board (CHREB) at the University of Calgary (REB21-0610), and the study adhered to the guidelines established by the Declaration of Helsinki.

Healthy, nonsmoking individuals defined as female-sex assigned at birth, residing in Canada with access to the internet and an iPhone, were recruited based on their self-reported average MC length ≥21 and ≤35 days. Participants were assumed to be naturally cycling, but ovulation and hormone values were not verified at the time of enrollment^18^ (see Box 1 for definition). Other eligibility criteria included ages 18–35 years, absence of hormonal contraceptives, hormonal therapies, pregnancy, or lactation 6 months prior to the study start. Exclusion criteria included nonhormonal intrauterine device implantation in the previous 6 months, history of reproductive surgery, presence of chronic disease(s) impacting hormone production, and current drug use that may interfere with steroid hormone metabolism.

**Box 1. Study inclusion definitions.**

1. **Healthy, naturally cycling female**^18^—free from chronic disease, conditions, or comorbidities known to impact the MC; female-sexed individuals who experience menstruation, with MC lengths ≥21 and ≤35 days, but without confirmed ovulation (ovulation was not confirmed by urinary LH surge or verified by hormone concentrations *via* blood sample analysis).
2. **Eumenorrheic cycle**^18^—MC lengths ≥21 and ≤35 days resulting in nine or more consecutive periods per year, plus evidence of LH surge, plus correct hormonal profile, plus no hormonal contraceptive use 3 months prior to recruitment.
3. **Menstruation**^7,18^—The start and end of vaginal bleeding as a self-reported symptom of menses, indicating a hormonal change of low estradiol and progesterone and the shedding of the uterine lining.

Participants were asked to download the app and enroll on an iPhone so they could set up an account prior to the study protocol start. A unique study identification was assigned to each participant, which they used to register as a research participant in the app (version 2.0.8–2.1.7). This was accompanied by a participant code granting full access to the premium subscription of the mobile app for 6 months. Participants were asked to self-report menstruation (see Box 1 for definition) start date, cycle end date, and answer one personal insight question daily in the app for the duration of the study. App reminders were sent to participants up to 14 days after the expected menstruation start day to review and confirm or adjust their expected cycle start date.

The m3stepMC tracking method was employed to determine the menstrual phases by (1) calendar counting to map the start (*i.e.*, day 1) and end of menstruation (see Box 1 for definition) and cycle length, (2) Clearblue Digital Ovulation Test Kits (Swiss Precision Diagnostics GmbH, Geneva, Switzerland) to identify the LH surge day, and (3) salivary hormone kits from Doctor’s Data (DD), formerly Labrix Lab (DDI., St. Charles, IL), to identify the estimated mid-luteal phase. In the first recorded cycle, participants self-reported cycle menstruation dates, and this was continued for the duration of the protocol. During the second recorded cycle, participants began ovulation testing on cycle day 8^2,12,19^ and continued until either a positive test result or until 10 test strips were used. In the third cycle, a salivary collection day was assigned to participants with a positive ovulation test from cycle two. The assigned salivary collection day was determined by taking the positive ovulation test day from the second MC (*i.e.*, LH surge day indicated by Clearblue Digital Ovulation Test Kits) and adding 8 days.^2,19^ For example, if a participant had a positive ovulation test on cycle day 10 in their second MC, the salivary collection day would be on cycle day 18 of the third MC. All study data for the m3stepMC tracking method were collected and managed using REDCap—a secure, web-based electronic database tool—hosted at the University of Calgary, Canada.^20,21^ Weekly and monthly follow-up email reminders were sent to participants from the research coordinator to complete the study protocol.

### Statistical analysis

The analysis included eumenorrheic cycles (see Table 1 for comprehensive definition) defined as a cycle length ≥21 and ≤35 days, a positive LH surge test having occurred at time of testing, and salivary hormones within the provided DD’s reference intervals (Estradiol = 0.6–4.5 pg/mL; Progesterone = 127.0–446.0 pg/mL). The analysis plan involved assessing the agreement between measurement methods with raw data regardless of cycle date confirmation in the app. This meant that all cycle dates from the app were included but not confirmed by the calendar-counting method. Following completion of the data collection and analysis of the raw data, unconfirmed participants’ cycle dates in the app were identified and confirmed by the dates reported from the participant’s calendar-counting method. The unconfirmed cycle dates in the app were then adjusted to match the calendar-counting dates. The adjusted cycle dates in the app confirmed by the calendar-counting method updated the app-identified phases. The updated app phase identification data were then analyzed by assessing them with the m3stepMC method-identified phases. These results are reported in this study.

**Table 1.** Menstrual Cycle Phases, Menstrual Events, and Definitions Identified by the m3stepMC Tracking Method and App

Phase name of the m3stepMC tracking method	Phase definition of m3stepMC tracking method	Menstrual events captured	Phase identified in the app
Estimated start of mid-follicular phase	LH surge day minus 7 days.	Menstruation/low hormones has ended; estradiol levels are about to start increasing.	End of phase one
LH surge day	Cycle day LH surge occurred verified by urinary ovulation kits.	Estradiol has peaked, signaling the release of LH.	End of phase two
Estimated end of 48-hour ovulatory window	LH surge day plus 48 hours (*i.e.*, 2 days).	Ovulation has likely occurred, and estradiol and progesterone levels begin to increase.	Midpoint of phase three
Estimated mid-luteal phase	Cycle day salivary testing occurred with hormone levels suggestive of mid-luteal range; verified by hormone values within range (Estradiol: 0.6–4.5 pg/mL; Progesterone: 127–446 pg/mL).	If ovulation has occurred, estradiol and progesterone levels are likely to peak.	Midpoint of phase four
Estimated start of late-luteal phase	Estimated mid-luteal phase day verified by hormone values plus 4 days.	Estradiol and progesterone levels have partially decreased and are close to baseline levels.	Start of phase five

App-based phase identifications are dynamic to the overall cycle length for a given cycle.

LH, luteinizing hormone; m3stepMC, modified, three-step MC; MC, menstrual cycle.

All descriptive data are presented as means (range), standard deviations (SD, ±), and frequency (%). Statistics and graphical analysis were performed using Microsoft Excel 2023 (Version 2310, Build 16.0.16924.20054) and STATA v. 17.0 (StataCorp. 2021. *Stata Statistical Software: Release 17*. College Station, TX: StataCorp LLC). Visual histogram inspections were used to assess variables for normality. Bland–Altman plots^22^ were used to assess the agreement between measurement methods per phase identified in the app and reported as mean bias, SD (±), 95% confidence interval (CI), and 95% limits of agreement (LoA). To assist with the agreement analysis between measurement methods (the m3stepMC tracking method relies on a single day, and the app uses >1 day), a single day was selected from each phase identified by the app to approximate the MC events identified by the evidence-based method. The compared phase definitions and the menstrual events captured for the level of agreement analysis are described in Table 1. Pearson’s correlations (*r*) were completed between variables per phase to establish effect sizes of the results.

## Results

**FIG. 1.**
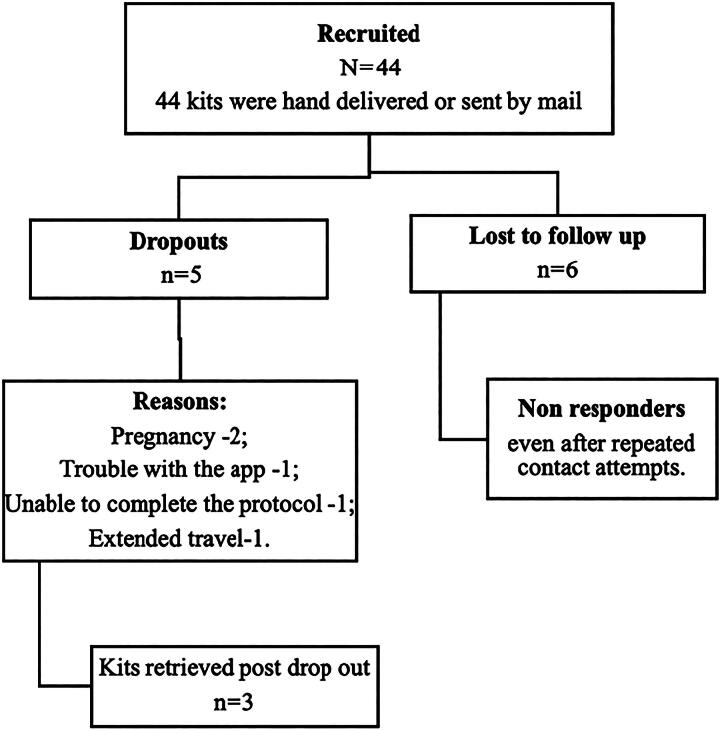
Overview of participant recruitment (*N* = 44).

A total of 44 participants were recruited, and 44 kits were hand-delivered or mailed to participants (Figure 1 for an overview of the recruitment and Table 2 for participant locations). Only three study kits were retrieved post-participant dropout. Five participants dropped out of the study because of pregnancy (2), trouble with the app (1), unable to complete the protocol (1), or extended travel (1), resulting in an 11% dropout rate (5/44). An additional six participants were lost to follow-up despite repeated attempts to contact them, resulting in a nonresponse rate of 14% (6/44). These participants (11) who dropped out or were lost to follow-up resided in Alberta (9), Ontario (1), and Nova Scotia (1).

**Table 2.** Demographics by Location, Ethnicity, and Menstrual Tracking Awareness in Eumenorrheic Females (Frequency, %)

Characteristics	Frequency, %
Location, *n* = 25	
Alberta	13, 52.0
British Columbia	4, 16.0
Saskatchewan	1, 4.0
Ontario	4, 16.0
Nova Scotia	3, 12.0
Ethnicity and cultural origin classification,^a^ *n* = 25	
North American	15, 60.0
European	7, 28.0
Other (Asian, Central or South American, or Latin)	3, 12.0
Menstrual tracking methods, *n* = 9	
Calendar counting	3, 12.0
Cervical mucus	3, 12.0
Ovulation tests	2, 8.0
Basal body temperature	1, 4.0

^a^ Classification based on the Statistics Canada list of cultural or ethnic origins (2021).

Other exclusions included a cycle length outside of 21–35 days (3), negative ovulation results (3), and app account registration error resulting in missing app data (2). Twenty-five remained with no missing physiological data from the m3stepMC method. and 11 had registered app accounts but had missing app data confirmations. Those with missing app data confirmations had their cycle dates confirmed and adjusted using the calendar-counting method. This resulted in a 56% completion rate from those recruited (25/44). The analysis for the estimated mid-follicular phase start, LH surge day, and estimated end of the 48-hour ovulatory window included these 25 participants who had an ovulatory cycle (*i.e.*, cycle length within 21–35 days with positive LH surge detected). However, eumenorrhea could not be verified using salivary hormones in 64% (16/25) of participants. The remaining 36% (9/25) who fit the eumenorrheic criteria (*i.e.*, cycle length within 21–35 days, positive LH surge in cycle two, and salivary hormones within DD’s reference intervals in cycle three) were used for the analysis of the final two phases—the estimated mid-luteal phase and estimated late-luteal phase midpoint.

The participants’ mean (SD, ±; range) age was 29.3 ± 4.24 years (18.00–36.00; 25), with a cycle length of 27.3 ± 2.38 days (23.00–34.00; *n*_cycles (25 + 9)_ = 34). Most participants were from Western Canada (72%), identified as North American (60.0%), and, at enrollment, reported having used an app before (88.0%). Only a third of participants (36.0%) were familiar with other methods of MC tracking (Table 2).

### Menstrual cycle characteristics

The mean day of detection for the LH surge using the ovulation kits was 14.2 ± 2.20 (10.00–19.00; 25) cycle day. Of those 25 participants, 16 (64%) did not meet the salivary hormone reference intervals, were potentially indicative of luteal phase deficiency, were anovulatory in cycle three, or the m3stepMC method did not capture individual variability within the mid-luteal phase.^23^ For the remaining nine participants, salivary hormones were collected at the estimated mid-luteal phase on cycle day 22.5 ± 2.51 (19.00–27.00; 9), with estradiol and progesterone concentration levels of 1.4 *±* 0.42 pg/mL (0.80–1.90) and 189.1 *±* 52.33 pg/mL (137.00–302.00), respectively.

### Level of agreement

The results from the agreement analysis for each phase identified are summarized in Table 3.

**Table 3.** Mean (SD, ±), Differences (Days), 95% CI (Days), Lower and Upper LoA (Days), and Correlations from the Level of Agreement of Phases Identified Between the m3stepMC Tracking Method and the App

Phase identified	*n*	Mean bias ± days	95% CI days	Lower LoA days	Upper LoA days	Correlation (*r*)
Estimated mid-follicular start day to app phase one	25	0.6 ± 1.66	−0.05 to 1.25	−2.65	3.85	0.66
LH surge day to app phase two	25	−0.6 ± 1.71	−1.27 to 0.07	−3.95	2.75	0.64
Estimated end of 48-hour ovulatory window day to app phase three	25	−0.6 ± 1.71	−1.27 to 0.07	−3.95	2.75	0.64
Estimated mid-luteal phase day to app phase four	9	−2.2 ± 0.97	−3.00 to −2.00	−4.13	−0.32	0.94
Estimated late-luteal midpoint day to app phase five	9	−2.2 ± 0.97	−3.00 to −2.00	−4.13	−0.32	0.94

CI, confidence interval; LoA, limit of agreement; SD, standard deviation.

**FIG. 2.**
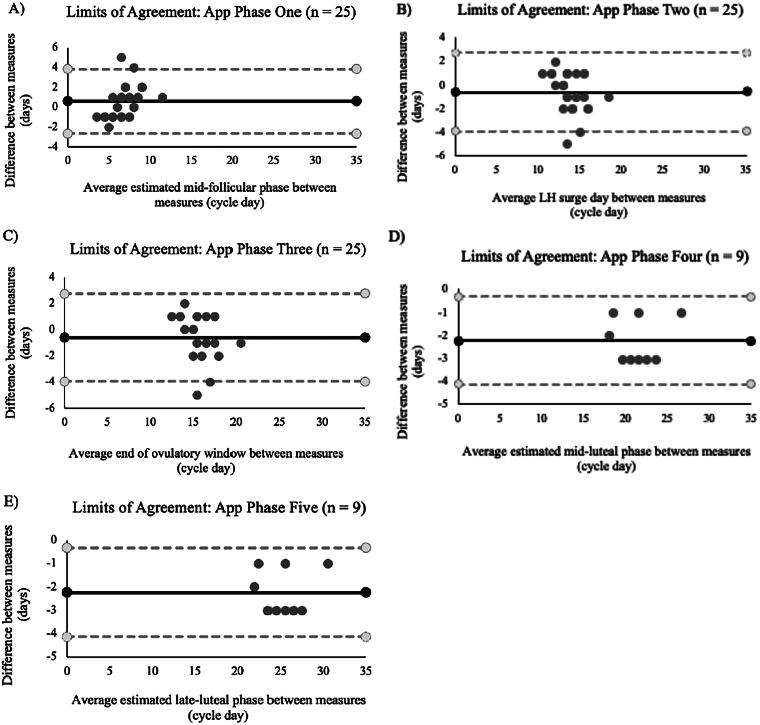
Limits of agreement of identified menstrual cycle day between the **(A)** estimated end of the mid-follicular phase by urinary ovulation tests minus 7 days and the end of app phase one (*n* = 25), **(B)** luteinizing hormone surge day measured by urinary ovulation tests and the end of app phase two (*n* = 25), **(C)** estimated end of the ovulatory window between 48 hours post-luteinizing hormone surge measured *via* urinary ovulation tests and the midpoint of app phase three (*n* = 25), **(D)** estimated mid-luteal phase between salivary hormone testing day verified by hormone levels and the end of app phase four (*n* = 9), and **(E)** the estimated late-luteal phase between salivary hormone testing day plus 4 days (verified by hormone levels) and the start of app phase five (*n* = 9).

The agreement of the estimated mid-follicular phase identified by the app’s end of phase one to the estimated mid-follicular start day using urinary ovulation tests had a bias of 0.6 ± 1.66 days (95% CI: −0.05 to 1.25), with a lower LoA of −2.65 days and an upper LoA of 3.85 days (Fig. 2A). The positive bias indicates that the average app-estimated mid-follicular start day was 0.6 days after the m3stepMC tracking method-identified day. The SD showed a range of differences of *±*1.66 days and the LoA suggests that 95% of the differences between measures are likely to land between <3 days prior (−2.65) or up to 4 days later (3.85) than the day identified using the m3stepMC tracking method.

The agreement of the estimated LH surge day identified by the app’s end of phase two to the direct measurement of the LH surge (urinary ovulation test) had a bias of −0.6 ± 1.71 days (95% CI: −1.27 to 0.07), with a lower LoA of −3.95 days and an upper LoA of 2.75 days (Fig. 2B). These results were the same for the estimated 48-hour ovulatory window (Fig. 2C). The negative bias indicates that the average identified LH surge day by the app was 0.6 days before the m3stepMC tracking method-identified LH surge day and, subsequently, the end of the 48-hour ovulatory window. The SD showed a range of differences up to 3 days (±1.71), and the LoA suggests that 95% of the differences between measures are likely to land between <4 days prior (−3.95) or up to 3 days later (2.75) than the m3stepMC method-detected LH surge.

The agreement of the estimated mid-luteal phase day identified by the app’s midpoint of phase four to salivary hormone tests had a bias of −2.2 ± 0.97 days (95% CI: −3.00 to −2.00), with a lower LoA of −4.13 days and an upper LoA of −0.32 days (Fig. 2D). These results were the same for the estimated late-luteal phase midpoint day to the start of phase five identified by the app (Fig. 2E). The negative bias indicates that the average estimated mid-luteal phase day identified by the app was 2.2 days before the m3stepMC tracking method-identified mid-luteal phase day and subsequently, the estimated late-luteal phase midpoint day. The SD showed a range of differences up to 2 days (±0.97) while 95% of the differences between measures were likely to land between less than one (−0.32) and up to 5 days prior (−4.13) to the estimated mid-luteal phase and the estimated late-luteal phase midpoint day identified by the m3stepMC method.

## Discussion

An important feature in apps that track the MC is the precise identification of the LH surge and the ovulatory or fertile window to distinguish between phases (follicular, luteal) and, subsequently, reduce the burden of direct LH hormone testing.^3,4^ The results of this study describe the app’s phase classification in 25 ovulatory cycles to identify the estimated start of the mid-follicular phase, the LH surge day, and the end of the estimated 48-hour ovulatory window. When examining the estimated mid-luteal phase and estimated midpoint of the late-luteal phase, the results describe the app’s phase classification in 9 participants. The estimated mid-follicular phase (0.6), LH surge day (−0.6), and the end of the estimated 48-hour ovulatory window (−0.6) demonstrate that the average estimated day identified in these phases by the app had a difference within 1 day. The mid-follicular phase can range from 3 to 7 days prior to the LH surge, providing a source of MC variability within and between individuals.^19^ The variability in the LH surge day found in a previous study using direct hormone measurement methods between cycles within an individual was 3.9 ± 3.7 days (95% CI: 1–13 days), and between individuals 6.6 days.^2^ Ovulation has been reported to occur 24 hours after the LH surge in 51% of cycles, in 48 hours after the LH surge in 43% of cycles,^24^ or within a 2-day period around the identified LH peak 78.2% of the time.^25^ These previous studies show the variability that occurs in MCs within and between individuals, which this study also observed.

The results reported for the app’s estimated mid-luteal phase and estimated midpoint of the late-luteal phase in nine eumenorrheic cycles were different (−2.2) compared with the estimated mid-follicular phase, LH surge day, and end of the estimated 48-hour ovulatory window. A difference >2 days (−2.2) between the m3stepMC tracking method and the app suggests that the app may be identifying these latter phases slightly earlier than the m3stepMC method. A contributing reason for these results includes the measurement of salivary hormones occurring on a single day based on the previous cycle’s ovulation date, resulting in an estimated mid-luteal phase day. It is possible for the mid-luteal phase to last several days when using reference ranges or cut-off values, according to Ecochard et al.,^23^ and who previously reported progesterone levels during the mid-luteal phase with mean length of 5.6 ± 3 days. Furthermore, individual cycle variability can add to the challenge of capturing the mid-luteal phase, as seen in the well-known three-step verification method where the mid-luteal phase was only captured in 70% of naturally menstruating participants.^2^ These nuances are important to understand given the level of variability noted in the literature^8–10^ and, subsequently, observed in our population with strict inclusion criteria.

Primary analysis assessed the agreement between measurement methods with raw data. Raw data included all cycle dates that were either confirmed or unconfirmed in the app to assess real-time input and demonstrate implications associated with unconfirmed data in apps that track the MC.^3,26^ Following analysis of the raw data, the adjusted app phase identifications confirmed by the calendar-counting data were analyzed. The results using the adjusted app dates and phases were reported in the present study to describe the baseline agreement between measurement methods when app data are confirmed. The bias reported for each predefined app phase when cycle dates are confirmed is defined as the average difference or agreement between measurement methods. For individuals with less cycle variability, the bias may be near zero, meaning less difference between measurement methods. The opposite—a bias further away from zero suggesting a greater difference between methods—may occur in those with greater cycle variability. For example, it was observed in this dataset that the further away the LH surge occurred from the midpoint of the cycle relative to cycle length, the greater the difference was between measurement methods, resulting in a bias further away from zero. The remaining users whose LH surge occurred at the approximate midpoint of their cycle had a bias closer to zero. This observation was outside of the present study objective but may be a consideration for future research.

It is important to consider the range of differences demonstrated through the SD and LoA for all five predefined phases when interpreting these results. The SD and LoA values suggest that there may be a range of differences between the estimated day identified in the app and the m3stepMC tracking method. Sources of cycle variability that may have influenced the results in this study include an LH surge that did not occur near the midpoint of the cycle and variability in mid-luteal phase length. Cycle variability is a common occurrence in individuals with natural MCs and can be influenced by various life events such as physical or psychological stress.^27^ These life events are generally not well accounted for when reviewing the current literature on MC tracking methods, which could influence the SD and LoA in addition to the occurrence of normal cycle variability.^28,29^

The study completion rate was 56% (25/44). The remaining 44% of participants either dropped out (5), were lost to follow-up (6), not considered eumenorrheic (8), or did not create an account in the app during the protocol lead up to the study start (2). Those that did not create an app account were recorded as missing data; however, they did complete the m3stepMC method (both met the inclusion criteria with a positive ovulation test, and one met DD’s salivary reference intervals). Further, approximately 30% of the participants were unable to comply with the study protocol (13/44). Three participants were excluded based on cycle lengths >35 days in duration and had negative ovulation results. The cycle lengths that occurred during the study protocol of the three excluded participants were generally greater than the average cycle lengths reported at baseline. For example, one participant had an average baseline cycle length of 29 days, yet their cycle length was 40 days in cycle one, 39 days in cycle two, and 37 days in cycle three. The other two participants had noticeable jumps in one cycle length compared with the other two cycles, and this is illustrated by a cycle length increasing from 35 to 57 days. Interestingly, this same participant was anovulatory in cycle two, yet met DD’s salivary reference intervals in the 57-day cycle. Those who were excluded based on ovulation status (3) had salivary hormones outside of the reference intervals but cycle lengths between 26 and 32 days. These details of the eight who did not meet the eumenorrheic definition in our sample identify the variability that can occur between natural cycles.^8–10^

The Pearson’s *r* value suggested a strong correlation between the variables used for the level of agreement, resulting in a large effect size, suggesting the results have significance in practical, real-world settings. Therefore, a strength of this study was that it was pragmatic in nature, demonstrating real-world data for both app use and completion of urinary ovulation and salivary testing measures (*i.e.*, no lab visits were required). The nature of the study design allowed recruitment to take place across Canada over a large demographic, increasing generalizability. Measures for calendar counting and MC length were prospectively self-reported in the app, and this could be a limitation. However, Shea et al.^30^ compared app-tracked data with self-reported data and found that 44% of participants recalled their exact period length and 83% recalled it within ±1 day, suggesting that potential recall bias may be negligible.

The demographic information collected for cultural or ethnic background used the Statistics Canada (2021) classification, which included a category of “North American.” The ethnicity of participants who selected this category is unclear given the ethnic diversity within North America. This may be important as some studies suggest that cycle variability is different between ethnicities with Asians and Hispanics having the largest variability.^31^ The app was only available on iPhone devices, which impacted accessibility for the greater community of mobile device users. For example, nine individuals were excluded from participating in this study due to not having access to an iPhone. Although our study was conducted in Canada, a study from the United Kingdom examined the demographic and personality diversity between iPhone and Android users and found differences between smartphone groups.^32^ The authors concluded that incorporating both iPhone and Android applications for data-collection purposes may mitigate any impact of potential individual differences.^32^

Data collected from a national Canadian survey reported that 43.7% of females aged 15–49 years used oral contraceptives, with an additional 4.3% using intrauterine devices, 2.4% using injections, and 1.8% using contraceptive patches or vaginal rings.^33^ As of 2023, it has been reported that Canada has one of the highest overall contraceptive use rates (73%) in women aged 15–49 years.^34^ Naturally cycling individuals are partially defined as not being on hormonal contraceptives for a minimum of 3 months.^18^ Some studies have used criteria of up to and extending beyond 6 months for an individual’s MC to return to normal after exposure to hormonal contraceptives, medications, or conditions impacting the MC such as pregnancy and breastfeeding.^8,14,35–37^ Due to the nature of the research question and specific population examined, this study employed the conservative approach of 6 months for participants. The strict inclusion of naturally cycling individuals likely influenced the recruitment numbers for the study. From these naturally cycling individuals, the results of this study only involved those with ovulatory cycles (*n* = 25) and, subsequently, eumenorrheic cycles when verified with salivary hormones (*n* = 9). This demonstrates that eumenorrheic cycles were in the minority (27.3%; 9/33) of our sample even with strict inclusion parameters and suggests that the study results may not be representative of the variability that may occur in the larger menstruating population.^8,9^

The observed prevalence of anovulation can range up to 18.6% in naturally cycling females.^37^ If ovulation status had been identified at the time of enrollment, the need to exclude participants based on negative ovulation status (12%; 4/33 after dropouts and loss to follow-up) may have been minimized. This could have impacted the number of full datasets included in our analysis. Furthermore, the burden of study duration (a minimum of 3 months) may have contributed to those who dropped out (5) and were lost to follow-up (6). Despite multiple follow-ups, other reasons for lack of retention in these participants (6) remain unknown.

Luteal phase deficiency was not assessed as an inclusion criterion; however, Schliep et al.^38^ found luteal phase deficiency defined by either a luteal phase duration of <10 days or suboptimal serum progesterone (<5.0 ng/mL) affected 8.9% and 8.4% of ovulatory cycles, respectively. When examining recreationally active women, Schaumberg et al.^2^ reported that 30% of participants with positive ovulation tests were potentially luteal-phase deficient based on suboptimal serum progesterone (<6.0 ng/mL). Despite confirmation of an LH surge, it is possible that some of the population recruited for this study may have experienced luteal phase deficiency, contributing to the reduced sample for the phases four and five analysis. Therefore, future studies should consider assessing luteal phase deficiency at enrollment if the aim of the study is to examine eumenorrheic individuals.^12^ Future research may also consider integrating LH surge tests as part of the inclusion criteria and combining ovulation and salivary tests in a single month to shorten time burden, potentially reducing participant dropout and loss-to-follow-up rate.

Ten test strips were provided for ovulation testing, and participants were classified as “negative” if a positive result was not indicated. Previous literature has stated that one of the largest sources of individual MC variability occurs in the follicular phase length, ranging from 10 to 22 days.^39^ The m3stepMC method used in this study assumed that ovulation would occur near the midpoint of the cycle but did not consider participants who may have had a longer cycle length and subsequent follicular phase. If a participant with a longer follicular phase ovulated after the 10 tests were completed, ovulation status may have been incorrectly labeled, potentially reducing the sample. Second, ovulation and salivary testing were not completed in the same cycle and may have contributed to measurement error in the estimated mid-luteal phase day identified by the m3stepMC method.^13^ Our lack of repeated measures in this study for ovulation and salivary testing may have limited the findings in Bland–Altman methods.^22,40^

Gold standards of serum hormone tests and transvaginal ultrasound were not employed due to costs.^6,41^ Although salivary hormones and urinary ovulation tests are reported to have positive comparability to gold standards,^15,42,43^ the m3stepMC method is not the current gold standard for hormone verification and ovulation detection. Therefore, validation to gold standards from the study results cannot be claimed and, subsequently, decreases generalizability.^6,24,35,41^

The app had five predefined MC phases based on the app’s phase classification system, which was developed prior to the study. Alignment of the m3stepMC tracking method to the predefined phases occurred after data collection, resulting in estimated values (*i.e.*, estimated start of mid-follicular phase, estimated end of 48-hour ovulatory window, estimated mid-luteal phase, and estimated late-luteal phase midpoint). To address this, a single day was selected for each phase estimate identified by the app to approximate and highlight MC events identified by the m3stepMC method. However, both measurement methods (*i.e.*, the m3stepMC method and the app) had limitations, which likely impact these results and identify that more work is needed in this area.

Drop-off in study participation is a common occurrence in mobile health app studies.^44–46^ In another MC tracking study where high app compliance was needed (≥80%), the number of cycles that met the inclusion criteria for analysis was 59.3% (708/1194 cycles).^26^ Similarly, this was observed in the present dataset, where 56.0% of participants (14/25) confirmed cycle start dates in the app. However, some participants (44.0%; 11/25) reported forgetting to record and confirm cycle dates in the app as the protocol progressed even with frequent app reminders and weekly and monthly follow-ups. This resulted in unconfirmed data and was addressed through retrospective app cycle data confirmation using the calendar-counting method.

## Conclusions

These pilot results describe the app’s estimated phase classifications compared with the estimated start of the mid-follicular phase, the directly measured LH surge day, the estimated end of the ovulatory window, the estimated mid-luteal phase, and the estimated late-luteal midpoint, respectively. The results are only generalizable to those with an iPhone and eumenorrheic cycles with cycle dates that are consistently entered and confirmed in the app.

Future opportunities to work with apps that track the MC under the broader spectrum of mHealth technologies continue to grow.^47^ This is likely due to a movement to understand the MC and its impacts,^48^ the increased uptake of MC apps,^17^ and the value put on scientific app accuracy.^47,49^ Several authors in the field of MC apps^50,51^ and mobile health development^52^ suggest that thorough app-researcher collaborations are needed to contribute to the literature in this area. While these results contribute to the literature, there remains a need for future collaborations that employ serial testing measures that include ovulation and hormone verification to objectively determine menstrual phases^12,53^ so as to remove the need for estimated values.

## Abbreviations Used

agreement: level of agreement
CHREB: Conjoint Health Research Ethics Board
CI: confidence interval
Est: estimated
LH: luteinizing hormone
LoA: limits of agreement
m3stepMC: modified, three-step MC
MC: menstrual cycle
*n*_cycles_: number of cycles
SD: standard deviation
the app: MC tracking app
